# Regulation of Ketone Body Metabolism and the Role of PPARα

**DOI:** 10.3390/ijms17122093

**Published:** 2016-12-13

**Authors:** Maja Grabacka, Malgorzata Pierzchalska, Matthew Dean, Krzysztof Reiss

**Affiliations:** 1 Department of Food Biotechnology, Faculty of Food Technology, University of Agriculture, ul. Balicka 122, 30-149 Kraków, Poland; m.pierzchalska@ur.krakow.pl; 2 Neurological Cancer Research, Stanley S. Scott Cancer Center, Louisiana State University Health Sciences Center, 1700 Tulane Ave, New Orleans, LA 70112, USA; mdean3@lsuhsc.edu (M.D.); kreiss@lsuhsc.edu (K.R.)

**Keywords:** β hydroxybutyrate, 3-hydroxy-3-methylglytaryl-CoA synthetase 2 (HMGCS2), fenofibrate, melanoma, glioma, fasting

## Abstract

Ketogenesis and ketolysis are central metabolic processes activated during the response to fasting. Ketogenesis is regulated in multiple stages, and a nuclear receptor peroxisome proliferator activated receptor α (PPARα) is one of the key transcription factors taking part in this regulation. PPARα is an important element in the metabolic network, where it participates in signaling driven by the main nutrient sensors, such as AMP-activated protein kinase (AMPK), PPARγ coactivator 1α (PGC-1α), and mammalian (mechanistic) target of rapamycin (mTOR) and induces hormonal mediators, such as fibroblast growth factor 21 (FGF21). This work describes the regulation of ketogenesis and ketolysis in normal and malignant cells and briefly summarizes the positive effects of ketone bodies in various neuropathologic conditions.

## 1. Introduction

Adaptation to limited nutritional resources in the environment requires the development of mechanisms that enable temporal functioning in a state of energy deficiency at both systemic and cellular levels. Different molecular and cellular mechanisms have evolved allowing survival during nutrient insufficiency. Some rely on the decrease of metabolic rates, body temperature, or even shutting down most of the live functions during deep hibernation, aestivation or brumation. Other strategies require development of metabolic flexibility and effective fuel management. Peroxisome Proliferator Activated Receptors (PPARs) are important regulators of cellular responses to variable nutrient supply during both fed and fasted states. Acting as transcription factors, and directly modulated by fatty acids and their derivatives, PPARs induce transcription of the proper set of genes, encoding proteins and enzymes indispensable for lipid, amino acid and carbohydrate metabolism. In this review, we make an attempt to outline the regulation of ketone body synthesis and utilization in normal and transformed cells, as well as summarize the role of PPARα in these processes.

## 2. Ketogenesis and Ketolysis

Metabolic adaptation to prolonged fasting in humans is based both on coordinated responses of vital organs, mainly liver, kidneys and muscles, and on restoring nutritional preferences at the cellular level. In the fed state, cells primarily rely on glucose metabolism, whereas during longer food deprivation blood glucose levels drop because glycogen reserves are only sufficient for less than a day. In such conditions, glucose is spared mainly for neurons, but also for erythrocytes and proliferating cells in bone marrow or those involved in tissue regeneration. The most important change in the systemic metabolism during fasting is triggered by glucagon and involves the mobilization of lipids stored in adipose tissue and break down of triglycerides to free fatty acids and glycerol [1,2]. These two components are subsequently catabolized in liver: fatty acids undergo β-oxidation to produce acetyl-CoA and glycerol is a substrate for glucose synthesis via gluconeogenesis. Acetyl-CoA enters the tricarboxylic acid cycle (TCA) cycle, where it condenses with oxaloacetate to form citrate, and is then oxidized to CO_2_. The high rate of fatty acid oxidation observed in the liver during starvation drives acetyl-CoA flux that exceeds the capacity of citrate synthesis, and the surplus becomes the substrate for ketogenesis. Peripheral tissues switch their metabolism to oxidation of fatty acid and ketone bodies, the latter being a very efficient fuel that is preferable even to glucose [3].

**Figure 1:**
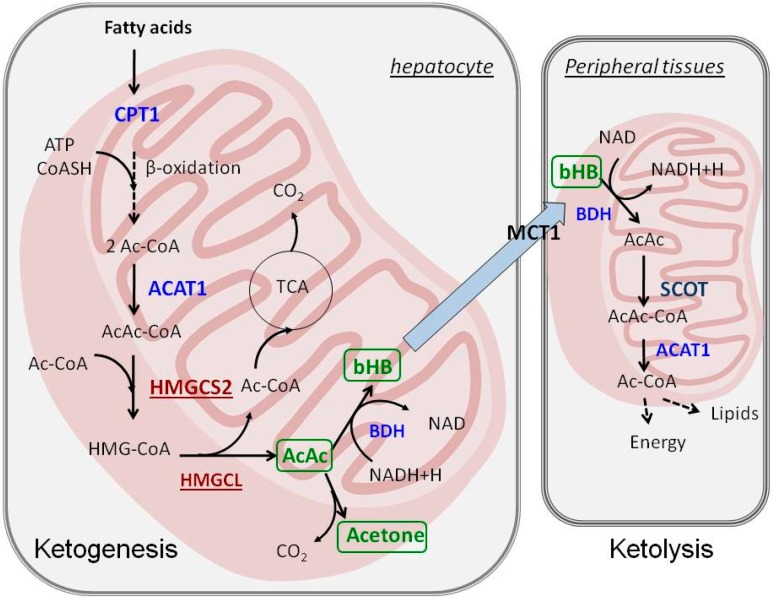
The metabolism of ketone bodies: ketogenesis takes place in hepatocyte mitochondria, whereas ketolysis involves utilization of ketone bodies in the mitochondria of peripheral tissues. ACAT1—acetoacetyl-CoA thiolase, Ac-CoA—acetyl-CoA, AcAc-CoA—acetoacetyl-CoA, BDH—β-hydroxybutyrate dehydrogenase, bHB—β-hydroxybutyrate, CPT1—carnitine palmitoyltransferase 1, HMGCL—HMG-CoA lyase, HMGCS2—HMG-CoA synthetase, MCT1—monocarboxylate transporter 1, SCOT—succinyl-CoA:3-ketocid-CoA transferase, TCA—tricarboxylic acid cycle.

The term “ketogenesis” defines a series of reactions that leads to the formation of so-called ketone bodies, which include β-hydroxybutyrate (bHB), acetoacetate and acetone. The process is primarily carried out in the mitochondria of hepatocytes, but kidney epithelia, astrocytes and enterocytes are also capable of, albeit to a lesser extent, producing ketone bodies. Ketogenesis requires efficient mitochondrial β-oxidation of fatty acids. Medium chain fatty acids, such as octanoate freely enter mitochondria and are readily broken down to acetyl-CoA [4]. However, long chain fatty acids, such as palmitate require carnitine-mediated transport to mitochondria through carnitine palmitoyltransferase (CPT1). CPT1 activity is regulated by the concentration of malonyl-CoA, an initial intermediate of fatty acid synthesis; therefore, CPT1 serves a regulatory node between fatty acid oxidation and biosynthesis [4]. Fatty acid oxidation product, acetyl-CoA is the substrate for ketogenesis and the first step involves condensation of two molecules of acetyl-CoA to form acetoacetyl-CoA in the reaction catalyzed by acetoacetyl-CoA thiolase (ACAT1, EC 2.3.1.9; Figure 1). Next, the third acetyl-CoA molecule is attached to form 3-hydroxy-3-methylglytaryl-CoA (HMG-CoA) by HMGCS2 (mitochondrial HMG-CoA synthetase, EC 2.3.3.10), which is the rate-limiting enzyme of the whole pathway. HMG-CoA is then transformed into the first type of ketone body, acetoacetate, and acetyl-CoA by HMG-CoA lyase (HMGCL, EC 4.1.3.4). The majority of newly formed acetoacetate is then reduced to bHB by NADH-dependent β-hydroxybutyrate dehydrogenase (BDH, EC 1.1.1.30). β-hydroxybutyrate is the most abundant ketone body in the circulation. The remaining fraction of acetoacetate in some tissues (such as lungs) is spontaneously decarboxylated into volatile acetone, the simplest ketone body. In fact, the presence of acetone in the air exhaled by diabetic patients is a symptom of a life threatening condition known as ketoacidosis.

Ketolysis is the opposite process to ketogenesis and encompasses a set of reactions that aim to regain energy via oxidation of ketone bodies, which takes place in mitochondria. In contrast to ketogenesis, which is carried out only in very specialized cells, almost all cells (except hepatocytes and majority of malignantly transformed cells) are capable of ketolysis. Ketone bodies (bHB and acetoacetate) are avidly absorbed from blood by the peripheral tissues by monocarboxylate transporter 1 (MCT1), which is expressed in virtually every cell. Acetoacetate, or bHB converted to acetoacetate by BDH, is converted next to acetoacetyl-CoA by a succinyl-CoA-dependent transferase (succinyl-CoA:3-ketoacid-CoA transferase, SCOT), which is the key reaction that enables ketone body utilization as energy substrates (Figure 1). In the next step, acetoacetyl-CoA is cleaved into two molecules of acetyl-CoA by ACAT1. Acetyl-CoA molecules are then oxidized in the TCA cycle and respiratory chain for ATP synthesis. Alternatively, under certain conditions, this acetoacetyl-CoA may be incorporated into lipids (cholesterol or fatty acids). The liver is the only tissue that does not express SCOT to prevent futile cycling of acetoacetate to HMG-CoA and vice versa.

## 3. Regulation of Ketogenesis—The Role of PPARα

Adaptation to prolonged fasting and starvation requires a thorough reprogramming of metabolism, which is regulated on four levels: (i) hormonal; (ii) transcriptional; (iii) by posttranslational modifications of key enzymes; and (iv) biochemical, i.e., substrate availability and allosteric effects. This categorization is somewhat artificial because processes that belong to one class tightly cooperate and are in a direct causal relationship with those from other classes.

### 3.1. Endocrine Regulation

The general principle of hormonal regulation states that anabolic hormones inhibit, and catabolic hormones stimulate ketogenesis [5]. Insulin, which is the main anabolic hormone, is principally important: in the presence of insulin ketogenesis is strongly inhibited, even when catabolic hormones are also secreted. Insulin acts in two complementary manners: first, it blocks lipolysis in adipocytes; and next, it promotes glucose uptake and oxidation by tissues, which results in elevated succinyl-CoA and malonyl-CoA levels. These intermediates are strong inhibitors of fatty acid oxidation and ketone body formation in liver and other ketogenic tissues. When insulin levels are low, the catabolic hormones, namely glucagon (secreted by the pancreas during hypoglycemia), as well as cortisol, growth hormone, catecholamines, epinephrine, norepinephrine, and thyroid hormones come into prominence [6,7]. They all stimulate lipolysis and the release of free fatty acids, as well as fatty acid transport to the liver and skeletal muscles. Increased influx of fatty acids to the liver induces their β-oxidation and subsequent ketogenesis. Among catabolic hormones, epinephrine and norepinephrine, which drive “fight or flight” reactions to stress, are particularly strong activators of lipolysis, fatty acid oxidation and ketogenesis, and remain active regardless of insulin levels [8,9].

### 3.2. Transcriptional Regulation

The mode of action of hormones and growth factors involves transcriptional reprogramming to provide expression of the proper enzymatic machinery for ketogenesis and ketolysis. PPARα is the chief transcription factor responsible for the induction of the majority of the genes necessary for fatty acid transport, uptake and oxidation, as well as ketone body biosynthesis and import [10,11,12,13,14,15]. PPARα is a nuclear receptor, whose endogenous ligands are fatty acids and their derivatives [16]. Transcriptional activation of PPARα target genes occurs, when heterodimeric complex of ligand-bound PPARα and retinoic X receptor (RXR) associate with peroxisome proliferator response elements (PPRE) in the gene promoters. PPARα-RXR complex recruits variety of co-activator proteins with a histone acetyltransferase activity, that belong to the CBP/p300 or SRC/p160 families (reviewed in [17]). This events enable chromatin remodeling and association of the general transcriptional complex with the promoters.

Observations from PPARα knockout mice emphasize its importance in coordinating cellular and systemic responses to fasting. These animals rapidly became hypoglycemic after food deprivation and fail to activate hepatic fatty acid oxidation and ketogenesis during fasting [15,18,19]. Among various endogenous PPARα agonists, long chain fatty acids and their derivatives such as acyl-CoA ester or acylethanoloamides, which are released through lipolysis and the mobilization of lipid stores, are particularly strong activators. Upon ligand binding PPARα transactivates genes encoding: fatty acid binding protein (FABP), carnitine palmitoyltransferase 1A (CPT1A), peroxisomal acyl-CoA oxidase, mitochondrial long and medium chain acyl-CoA dehydrogenases (LCAD, MCAD), as well as rate limiting enzyme of ketogenesis, mitochondrial 3-hydroxy-3-methylglutaryl-CoA synthase (HMGCS2). PPARα is necessary for launching the ketogenic transcriptional program, but HMGCS2 is a nodal point in the ketogenic pathway and is strictly controlled by other transcription factors and various posttranslational mechanisms.

The *Hmgcs2* gene contains the consensus peroxisome proliferator responsive element (PPRE) sequence localized at −104–92 bp in the promoter [20,21]. One of the most intriguing mechanisms of *Hmgcs2* transcription is the fact that HMGCS2 protein physically binds to PPARα and the complex enters the nucleus and transactivates the *Hmgcs2* gene through the PPRE [22]. HMGCS2 protein contains the canonical motif responsible for interaction with nuclear receptors, the so-called nuclear receptor interaction motif, LXXLL [23], which was expected to bind PPARα. Surprisingly, this motif is not necessary for the interaction, but the palmitoylation of some important cysteine residues (cysteines 166 and 305 in the human protein). Only palmitoylated HMGCS2 protein is able to bind PPARα and successfully transactivate its own transcription [24].

Besides PPARα, there are other transcription factors capable of impacting *Hmgcs2* transcription either positively or negatively. Examples of positive regulators include: CREB, SP1, COUP-TF and forkhead family—related transcription factors DKHRL1 and Foxa2 [21,25,26,27]. In opposition to these transcription factors, hepatocyte nuclear factor 4 (HNF4) represses Hmgcs2 transcription [28].

On the systemic level, ketogenesis is also regulated by monitoring the utilization of ketone bodies by peripheral tissues. During fasting, the main membrane transporter responsible for ketone body absorption, MCT1, is expressed at high levels, which enables efficient import and ketolysis. MCT1 is a ubiquitously expressed gene that contains PPRE in its promoter and is strongly transactivated by PPARα during fasting or in response to synthetic PPARα ligands [10].

### 3.3. Posttranslational Modifications

Palmitoylation of HMGCS2 is a particularly interesting example of protein posttranslational modification that influences both the interaction with other proteins, such as PPARα, and the subsequent regulation of transcription. This particular modification occurs spontaneously in a palmitoyl-CoA concentration dependent manner, and surprisingly, does not require transferase activity. First, the cysteine 166 is acylated by palmitoyl-CoA forming a thioester bond, and next, this acyl chain is transferred to the Cys 305 residue [24]. Importantly, acylation on this Cys residue of the acyl-chains >12 carbons is necessary for maintaining the physical interaction with PPARα protein and subsequent transactivation [24].

HMGCS2 activity is also regulated by acetylation and succinylation. These two modifications take place in mitochondria, and both result in inactivation of the enzyme. Acetylation of HMGCS2 occurs at Lys 310, 447 and 473 and is mediated by mitochondrial acetyltransferases. During fasting, a mitochondrial deacetylase, Sirt3, which belongs to the deacetylase/ADP-ribosylase family of sirtuins, removes acetyl groups and activates the enzyme [29]. In the same way, Sirt3 also activates the enzymes involved in fatty acid oxidation, such as LCAD [29], which contributes to the activation of ketogenesis in the liver. Sirt1 and Sirt3 cooperatively deacetylate cytoplasmic and mitochondrial proteins, respectively, and they seem to be a part of a general and evolutionary conserved mechanism of metabolic regulation, which can be found throughout the whole tree of life [30].

Apart from acetylation, succinylation is the second type of HMGCS2 modification that takes place in mitochondria and profoundly influences HMGCS2 activity. In contrast to acetylation, succinylation is a non-enzymatic, spontaneous process that depends on the available pool of succinyl-CoA. Succinylation on either cysteine or on lysine residues usually represses enzymatic activities. Early studies on the regulation of ketogenesis suggested that succinylation by succinyl-CoA is the primary process that results in enzyme inactivation in the liver in the presence of insulin. Elevation of glucagon levels, by hepatic perfusion or injections of rats with mannoheptulose, resulted in a significant increase in HMGCS2 activity and ketone body production. The studies by Lowe et al. [31] and Quant [32] revealed that the attachment of succinyl-CoA to the catalytic cysteine residue (Cys166) blocks the binding of acetoacetyl-CoA to the substrate. Glucagon, in addition to its positive effect on the delivery of free fatty acids to liver, also sharply decreased succinyl-CoA levels and HMGCS2 succinylation, which resulted in a strong activation of ketogenesis.

The detailed studies on mitochondrial protein succinylation performed by Eric Verdin’s group demonstrated the succinylation of the particular Lys residues of all the key enzymes involved in both ketogenesis (acetoacetyl-CoA thiolase, HMGCS2, HMGCL and BHD), and in fatty acid oxidation (acyl-CoA dehydrogenase family of proteins, trifunctional enzyme involved in β-oxidation: hydratase, oxidase and thiolase). Succinylation causes inactivation of those enzymes, and can be removed by the mitochondrial deacetylase/desuccinylase Sirt5 [33]. Importantly, HMGCS2 is the most heavily succinylated of these proteins. It contains 15 lysine residues that all could be potentially succinylated; however, succinylation of Lys83 and Lys310 triggers conformational changes within the active site and hinders the binding of acetoacetyl-CoA phosphate groups, which are necessary for catalysis. Both acetylation and succinylation engage Lys residues and, therefore, are mutually exclusive [33]. It remains to be elucidated how this competition affects overall HMGCS2 function.

### 3.4. Biochemical Regulation

The rate of ketogenesis depends on the velocity of HMG-CoA synthesis by HMGCS2. This reaction is performed in three steps: (1) cleavage of acetyl-CoA with formation of a covalent bond between the acetyl moiety and the thiol group of catalytic cysteine (acetyl-SH-Enzyme) with the release of free CoA-SH; (2) binding of acetoacetyl-CoA with acetyl-SH-Enzyme and formation of HMG-CoA (Enzyme-S-HMG-CoA); (3) hydrolysis of the HMG-CoA-Enzyme intermediate with the release of HMG-CoA and free Enzyme-SH [34]. This complex catalysis is classified as a bi-bi ping-pong mechanism. In such a case, the overall reaction rate depends on the concentrations of each substrate. Interestingly, the same substrates may also act as inhibitors when they are present within a certain range of concentrations [35]. A similar situation is true for the enzyme that catalyzes the first step of the ketogenesis pathway, acetoacetyl-CoA thiolase [36]. Therefore, the net rate of ketone body synthesis is very sensitive to the fluctuations in the concentration ratios of acetyl-CoA to acetoacetyl-CoA and acetyl-CoA to free CoA-SH. In particular, the process is slowed down when acetyl-CoA to CoA-SH ratio is low [35,36].

Ketogenesis also depends on the acetyl-CoA pool coming from fatty acid β-oxidation. Oxidation of long-chain fatty acids in mitochondria is controlled at the level of their transport by acylcarnitine transferase A (in the outer mitochondrial membrane) and B (in the inner membrane). Only the fatty acids of eight or fewer carbon atoms can freely enter into mitochondria. Carnitine palmitoyltransferase 1A activity is crucial for the supply of fatty acids, and this enzyme is reversibly blocked by malonyl-CoA, a fatty acid synthesis intermediate [37]. Therefore, in the fed state, when insulin-stimulated lipogenesis is occurring in the hepatocyte cytoplasm, fatty acid transport and their subsequent catabolism to ketone bodies in mitochondria is blocked.

It is important to note that fatty acids are the main, but not the exclusive source of acetyl-CoA for the production of ketone bodies. Ketogenesis can also be fueled by acetyl-CoA derived from catabolism of ketogenic amino acids: lysine, phenylalanine, tyrosine, tryptophan, isoleucine and leucine [38]. Experiments performed in rats revealed that the highest rate of ketogenic catabolism of lysine and leucine takes place in the kidneys, whereas tyrosine is avidly oxidized in the liver [39]. Leucine belongs to the group of branched chain amino acids (BCAA) together with isoleucine and valine, that all share a common catabolic pathway [40,41]. All three of these amino acids are essential (not synthesized by mammals) and are spared for the purpose of de novo protein synthesis, so they are not usually a significant source of acetyl-CoA for ketogenesis. Nevertheless, their catabolism to ketone bodies takes place during short overnight fasting [38], or under extreme conditions in which skeletal muscle wasting is involved such as chronic food deprivation, cancer cachexia or diabetic ketoacidosis [42,43].

Branched-chain ketoacid dehydrogenase (BCKD), the enzyme complex driving the BCAA catabolic flux is regulated in an allosteric manner. The complex consists of multiple protein subunits of three catalytic activities: BCKA decarboxylase (E1), dihydrolipoamide acyltransferase (E2) and dihydrolipoamide dehydrogenase (E3), and its function is regulated both by covalent modification and allosteric effectors [41]. A specific BCKD kinase phosphorylates E1 and inactivates the whole complex, which is also sensitive to allosteric inhibition by NADH and CoA esters (isobutyryl-CoA, methylbutyryl-CoA and isovaleryl-CoA—all three being the intermediates of the BCAA degradation pathway).

## 4. Nutrient-Responsive Intracellular Signaling in the Regulation of Ketogenesis

**Figure 2:**
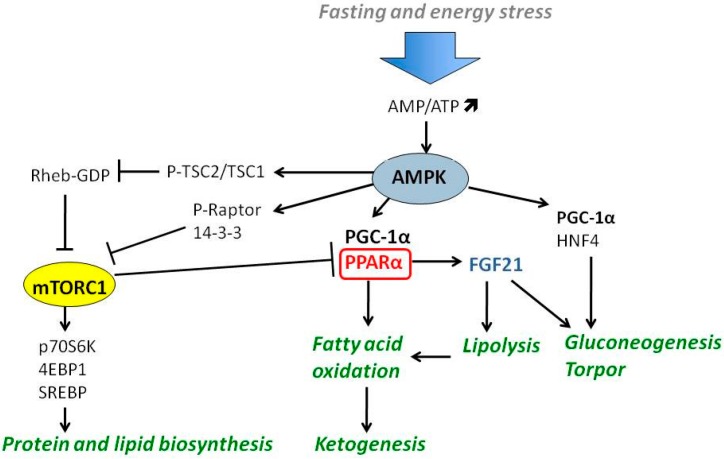
AMPK and mTOR complex 1 (mTORC1) respond to nutrient supply and cellular energy status. AMPK stimulates catabolism and ketogenesis through activation of PPARα and PGC-1α. mTORC1 blocks PPARα and induces anabolic processes, such as protein and lipid biosynthesis. The abbreviations are explained in the text.

Cellular adaptation to variable nutrient availability requires maintaining the balance between anabolic and catabolic processes to ensure proper ATP homeostasis. The network of interactions among signal transducing kinases, such as AMPK (AMP-activated protein kinase) and mammalian/mechanistic target of rapamycin (mTOR), and transcription factors or coactivators, such as PPARα and PGC-1α, is engaged in maintaining this homeostasis (Figure 2).

### 4.1. PGC-1α-PPARα-FGF21 Axis

The negative energy balance that develops during prolonged fasting also induces an endo-paracrine response that drives the systemic energy conservation program. The chief player in this process is fibroblast growth factor 21 (FGF21). The studies on mice revealed that FGF21 is mainly produced in the liver and its expression is strongly upregulated by PPARα [44,45], but additional PPARα -independent transactivation mechanisms also exist, and involve retinoid orphan receptors (RORs) [46]. FGF21 activates hepatic lipolysis and ketogenesis in hepatocytes, and in various mammalian species is the chief endocrine factor driving metabolic reprogramming during torpor, which includes inhibition of cell growth in size and cell proliferation to preserve energy reserves [47]. Simultaneously, FGF21 induces expression of PGC-1α, and hepatic gluconeogenesis but not glycogenolysis, which consequently spares hepatic glycogen reserves [48]. PGC-1α itself is an important coordinator of the starvation response and is responsible for induction of mitochondrial biogenesis. PGC-1α cooperates with PPARα and HNF4 in the process of stimulating fatty acid oxidation and gluconeogenesis, respectively [49,50,51]. It also controls ketolysis and ketone body utilization in peripheral tissues [52]. In contrast to mice, in humans FGF21 does not regulate an immediate starvation response and its blood level does not rise until 7–10 days of fasting, when bHB plasma levels are already 70-fold higher than in fed state [53,54]. This might be partially attributed to significantly higher metabolic rate observed in rodents comparing to humans, but also indicates that FGF21 is not a prime regulator of ketogenesis, but instead it drives late adaptive response to fasting, such as gluconeogenesis from amino acids derived from break down of tissues, such as muscle wasting [54]. Nevertheless, the cooperation among PPARα, PGC-1α and FGF21 (presented in the Figure 2) is necessary for the proper induction of expression of metabolic genes involved in the downstream responses to starvation.

### 4.2. The Role of AMPK and mTOR

Energy deficit at the cellular level is manifested by an increase in the AMP to ATP ratio. This occurs in hypoglycemia or ischemia, when glucose supply is insufficient, or in various stress conditions when ATP generation is inadequate compared to the needs, e.g., due to an impairment of the mitochondrial function. The major sensor protein that reacts to this deficit is AMPK. AMPK is activated by AMP and triggers a multidirectional rescue program aimed at maximization of ATP production accompanied by a simultaneous reduction of ATP expenditure on anabolic processes [55]. The latter is accomplished by phosphorylation and inactivation of the two AMPK canonical target proteins: acetyl-CoA carboxylase (ACC) and HMG-CoA reductase (HMGCR), the rate limiting enzymes of fatty acid and steroid synthesis, respectively [56]. Shutting down ACC activity decreases the concentration of its product, malonyl-CoA, and therefore releases CPT-1A and allows the efficient transport of fatty-acyl chains to mitochondria where they can subsequently undergo β-oxidation.

AMPK blocks anabolic processes such as protein biosynthesis, cell growth and proliferation by antagonizing mTOR. mTOR kinase operates in two distinct multiprotein complexes: (i) mTORC1 consisting of Raptor (regulatory-associated protein of mTOR), PRAS40 (proline-rich Akt substrate of 40 kDa), mLST8 and mTOR; and (ii) mTORC2 which includes Rictor, mSIN1, PRR5, mLST8 and mTOR [57]. The complexes differ in their response to nutrient-induced signaling and rapamycin sensitivity. mTORC1 is inhibited by rapamycin, as well as low levels of glucose, amino acids or oxygen. On the other hand, mTORC2 is fairly insensitive to these stimuli. Activity of both complexes is regulated tightly, but independently from each other, by a series of phosphorylation/dephosphorylation events driven by various kinases [58]. mTORC1 is engaged in the activation of nutrient and growth factor-stimulated protein biosynthesis, mainly by phosphorylation of two proteins: p70S6K1 and 4EBP1. Additionally, mTORC1 stimulates lipogenesis through the activation of sterol response element binding protein (SREBP) [59,60,61].

AMPK is one of the most prominent negative regulators of mTORC1. AMPK inhibits mTORC1 in two ways: (1) by a direct phosphorylation of Raptor on Ser722 and Ser792 residues; and (2) by phosphorylation of tumor suppressor tuberin (TSC2) on Ser1387 and Thr1271 residues. Phosphorylated Raptor binds 14-3-3 proteins and therefore is unable to form functional mTORC1 [62]. Phosphorylation of TSC2, a member of TSC1/TSC2 (hamartin–tuberin) complex, activates its GTPase activity that leads to inactivation of a small Ras-like G-protein Rheb that subsequently blocks mTORC1 activation [63,64]. By targeting mTORC1, AMPK acts as a metabolic checkpoint (Figure 2) [62], which under different energy deficient circumstances, arrests biosynthesis of macromolecules that are needed for cell cycle progression, and launches a rescue program to restore energy supplies.

Interestingly, mTORC1 inhibition in the liver is required for the activation of ketogenesis in response to fasting [65]. In normal mice capable of ketogenesis, mTORC1 activity is high in the fed but low in the fasted state. However, in animals with constitutively active hepatic mTORC1, ketogenesis does not occur due to the block of the PPARα-mediated expression of ketogenic genes. mTORC1 inhibition is also required for the dissociation of nuclear corepressor, NCoR1, and recruitment of transcriptional coactivators and histone acetyltransferases to the PPARα regulated promoters, which are all necessary to ensure efficient transcription [65]. Physiologically, mTORC1 inhibition is mostly driven by AMPK, so this is another example of cooperation between AMPK and PPARα in the activation of ketogenesis.

### 4.3. Ketone Bodies as Signaling Intermediates

Apart from their roles in indirectly mediating cellular responses through mechanisms involving AMPK and mTOR, more recently, ketone bodies have been shown to possess direct signaling capabilities of their own. There are multiple mechanisms through which ketone bodies have been shown to affect cellular signaling including; (1) directly modulating the activity of histone deacetylases (HDACs) and (2) activating particular G-protein coupled receptors (GPCRs).

Butyrate was one of the first metabolites discovered to directly bind to and inhibit HDAC activity [66]. More recently, bHB has also been shown to inhibit the activity of class I HDACs (HDACs 1, 2, 3, 8), although butyrate inhibits more efficiently [67]. This is significant because the role of HDACs is to deacetylate lysine residues of histone proteins [68]. This hypoacetylation of histones is associated with transcriptional repression, thus, the ultimate effect of bHB is histone hyperacetylation and an increase in transcription of target genes. Because HDACs are often found in complexes with other co-activators and co-repressors, the set of genes that can be modulated is extensive (HDAC1 knockout results in a seven percent change in global gene expression in mouse embryonic stem cells) [69]. In more recent studies, bHB inhibited HDACs 1, 3 and 4 in vitro with an IC_50_ of 2–5 mM [67], which is both attainable through ketogenic diets, prolonged fasting or exhaustive exercise (0.5–3 mM) [2,70,71] and free from the harm associated with pathogenic ketoacidosis (10–25 mM) [72]. In vitro treatment of cultured cells with bHB induces dose-dependent hyperacetylation of histones, which is similar to the in vivo hyperacetylation effects seen in the kidneys of mice following infusion of bHB via osmotic pump [67]. The full complement of genes that have altered expression in the presence of bHB remains to be determined, but several of them appear to be in the FOXO3a network and control processes involved in cell survival and oxidative stress responses [67]. These intriguing new roles for ketone bodies may reveal how these molecules exert their effects on both cellular processes and metabolism.

Epigenetic effects of both sodium butyrate and bHB exerted through the inhibition of HDAC3 are crucially important for regulation of FGF21 expression in newborn and adult mice [73,74]. This regulation is a vital part metabolic switch from intrauterine glucose catabolism to milk fatty acid oxidation that occurs in mammals after birth. This metabolic adaptation to suckling period begins in the late prenatal stage by glucocorticoid receptor driven expression of PPARα in fetal liver. PPARα triggers the expression genes involved in biogenesis of peroxisomes, peroxisomal and mitochondrial fatty acid oxidation [73]. These events clearly precede the induction of FGF21 expression, which takes place postnatally. FGF21 expression is in that case regulated not only by PPARα, but also by HDAC3-mediated repression. After birth, when newborn mice start to feed on milk, bHB blood concentration starts to increase and it inhibits HDAC3, and subsequently releases FGF21 promoter from HDAC repression. The de-repressed promoter is efficiently transactivated by PPARα [73]. Similarly, in adult mice, butyrate has also been shown to inhibit HDAC3 that resides in the FGF21 promoter and binds PPARα in the co-repressor complex [74]. HDAC3 inhibition releases PPARα, which is therefore able to induce FGF21 transcription. This leads to enhanced ketogenesis and fatty acid utilization [74].

In addition to the effects of ketone bodies on the HDACs, they have also been found to bind to specific G_i/o_ GPCRs. The first of these receptors was originally known as the nicotinic acid receptor, but has since be renamed the hydroxycarboxylic acid receptor 2 (HCAR2) in light of its ability to bind bHB. Activation of this receptor by bHB results in a decrease in intracellular cyclic AMP (cAMP) levels [75], which causes a decrease in lipolysis in adipocytes [76]. It is thought that this functions as a negative feedback loop during extended fasting and that it controls the rate of fatty acid mobilization such that fat stores are mobilized at a rate, at which they can be utilized but not wasted [77]. Although this receptor is expressed in the brain, the effects of bHB on its function in that tissue are not as well defined [78]. Since HCARs are also found on certain cells of the immune system such as macrophages and microglia, it is likely that ketone bodies modulate inflammatory responses. Indeed, there is experimental evidence for their effects on immune cells, which shows that bHB activates a subset of neuroprotective genes in macrophages [79]. Apart from the ability of bHB to activate HCAR2, it has also been shown to block the free fatty acid receptor 3 (FFAR3, GPCR41). Inhibition of this G_i/o_ receptor by bHB results in a decrease in propionate-induced cAMP production, with a concomitant decrease in activation of the ERK cascade. While FFAR3 is expressed in multiple tissues, in the brain, it enhances sympathetic nervous system activity, which results in increased metabolic rate that is blocked by increasing bHB concentrations [78]. These exciting new data reveal that ketone bodies have intrinsic signaling capacities that in some cases may impact cellular or organismal metabolism, and in others, affect processes such as inflammation, which are known to be involved in certain neurological conditions such as Alzheimer’s, Parkinson’s, and brain cancers [80,81,82]. This highlights the importance in further understanding the complex roles of ketone bodies in modulating cellular signaling responses.

## 5. Brain as an Example of Ketolytic Organ

**Figure 3:**
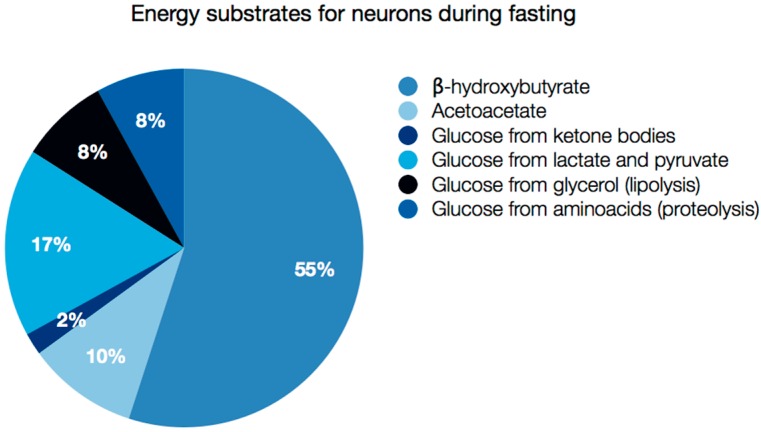
Energy substrates for brain during fasting. The values indicate the percentage of energy derived from utilization of each substrate.

Ketone body supply and metabolism is particularly important in the brain. In this tissue ketolysis accounts for between 60% and 70% of the energy supply during starvation (the majority of this fraction from bHB), and the rest, namely 30%–40% comes from glucose derived from various sources: amino acids, glycerol, lactate and pyruvate catabolism (Figure 3) [1,2]. Fatty acid oxidation, on the other hand, is not a source of ATP for neurons, because it takes place exclusively in astrocytes [83,84]. The experiments with rats subjected to ^13^C_4_-octanoate infusion through jugular vein revealed that astrocytes were able to derive about 20% of energy produced in brain from octanoate oxidation [85]. Medium chain fatty acids, such as octanoate are able to cross the blood-brain barrier (BBB) [86], but non-esterified long chain fatty acids (NEFA), which are present in the blood mostly as conjugates with albumin, cannot enter the brain at the rate sufficient to meet acute energy demands [87,88]. Astrocytic β-oxidation of long chain essential and nonessential fatty acids is therefore not so important for energy production, but indispensable for myelin maintenance [89]. Interestingly, blood profile of polyunsaturated fatty acids (PUFA) does not show strong correlation with fatty acid species present in the central nervous system [90], and brain cell membranes are significantly enriched in docosahexaenoic acid (DHA). The mechanism of long chain PUFA entry to the brain is still a matter of debate (as reviewed in [91]). The process most likely involves: (1) a passive diffusion and a flip-flop mechanism [92]; (2) protein-mediated transport through fatty acid transporters FATP 1-6, fatty acid translocase CD36 or caveolin-1 or Mfsd2a [91,93,94,95]; or transport as (3) a phospholipid constituent [96,97,98] or (4) in lipoproteins [99].

The difficulties in crossing BBB are no concern for ketone bodies, which are efficiently absorbed through monocarboxylate transporters (MCT1, MCT2), the expression of which increases significantly during fasting [100,101]. Additionally, enhanced β-oxidation of fatty acids might be dangerous for numerous reasons: (1) NEFA may lower the inner mitochondrial membrane potential, which slows down electron flux in respiratory chain and raises the risk of generating superoxide and other toxic reactive oxygen species (ROS); (2) the demand for high levels of oxygen needed for β-oxidation might exceed the actual oxygen concentrations in the brain, which leads to ineffective ATP production and the potential for hypoxia in metabolically active parts of brain parenchyma; (3) delivery and oxidation of fatty acids is too slow to satisfy ATP demands to sustain neuronal activity (as reviewed by Schonfeld and Reiser [102]).

Astrocytes, which retain the capability of β-oxidation, produce ketone bodies both from fatty acids and ketogenic amino acids, mostly leucine [103,104,105,106]. Like in hepatocytes, the ketogenic transcriptional program in astrocytes is driven by PPARα [107]. Therefore, apart from other metabolic substrates such as lactate [108,109] or glutamine [109], astrocytes also provide neurons with bHB, but they are a much less important source of ketone bodies than the hepatic pool delivered by the blood. Shuttling of glutamine from astrocytes to neurons, as a part of the so-called glutamate-glutamine cycle, enables the production and release of neurotransmitters: excitatory glutamate and inhibitory γ-aminobutyrate (GABA) by neurons [109,110]. Similarly to neurons, astrocytes are capable of very efficient ketolysis in ketotic states [83].

Ketolysis in the brain is able to sustain sufficient energy supply, because bHB is a very efficient source of energy in terms of ATP molecules produced per oxygen molecule, even compared to glucose [111]. Its unique property of increasing the redox potential between NADH/NAD (respiratory complex I) and ubiquinone/ubiquinon earned bHB a distinct name of “superfuel” [3,111,112].

Yudkoff and collaborators [113] suggested that ketosis and concomitant enhanced uptake and metabolism of bHB accelerate acetyl-CoA flux to the TCA cycle. After entering this pathway, acetyl-CoA reacts with oxaloacetate to form citrate. The high rate of TCA gradually reduces the pool of oxaloacetate, which limits transamination of glutamate to aspartate (by oxaloacetate/α-ketoglutarate aminotransferases). This in turn favors glutamate decarboxylation to GABA. Increased production of this inhibitory neurotransmitter likely contributes to the anti-convulsant and anti-epileptic effects of the ketogenic diet [113].

## 6. Neuroprotective and Therapeutic Activity of Ketone Bodies in Central Nervous System Pathologies

### 6.1. Epilepsy

The benefits of the ketogenic diet or fasting had been recognized much earlier than general knowledge about human metabolism during starvation was described. In 1920, refractory epilepsia in children was treated in such a way with considerable success [114,115]. Currently, the ketogenic diet is an adjunct therapy of choice, supporting the pharmacological treatment of epilepsy, or as a principal treatment method in drug-resistant cases, highlighting the efficacy in decreasing seizure frequency, especially in children [116,117]. It has been suggested that some anti-convulsive effects of the ketogenic diet might be attributed to PPARα activation by medium and long chain fatty acids [118]. This hypothesis is further supported by experimental evidence that a short-chain, branched fatty acid valproate and its numerous analogues, which are widely used as anti-epileptic drugs, indeed do activate PPARα [118,119]. Classical PPARα agonists, such as fenofibrate or Wy 14,643 have also been shown to alleviate nicotine-induced seizures in mice [120]. Interestingly, valproate effectively inhibits some members of HDAC class I and II [121,122,123], similarly to bHB [67]. Valproate administration and dietary interventions e.g., time-restricted feeding (TRF) that leads to increased bHB blood concentration both induce epigenetic modifications, such as histone hyperacetylation [124,125]. This might be an alternative mechanism of anti-epileptic activity of valproate and bHB. However, another potent HDAC inhibitor, trichostatin A, does not share anti-convulsant properties with valproate, which raises a question about HDAC involvement in pathology of epilepsia [126].

### 6.2. Neurodegenerative Diseases

Recently, a hypothesis on the beneficial effects of the ketogenic diet in other neurological disorders, such as Alzheimer’s Disease (AD) or Parkinson’s Disease (PD), has been widely discussed [127,128]. Clinical studies have revealed that medium chain triglycerides, an important component of the ketogenic diet, improve memory performance in patients with AD, which is exhibited in a positive correlation between blood bHB levels and improvement in Alzheimer’s Disease Assessment Scale-Cognitive Subscale (ADAS-cog) score [129]. Similarly, administration of AC-1202 compound, which consists of medium chain triglycerides used to induce a mild ketosis even in the presence of carbohydrates in diet, significantly improved the condition of AD patients with a higher risk of AD development (with the epsilon 4 variant of *ApoE* gene) compared to placebo [130]. A significant amelioration (based on assessment of Unified Parkinson’s Disease Rating Scale scores) was also recorded in PD patients, who voluntarily remained on the self-prepared ketogenic diet for four weeks [131].

### 6.3. Traumatic Brain Injuries

High levels of ketone bodies seem to be beneficial not only in the treatment of neurodegenerative diseases, but also in acute states after traumatic brain injuries (TBI). The results from animal studies on brain injury conditions, such as brain hypoxia and ischemia-reperfusion, showed that exogenous administration of bHB reduces brain damage and improves neuronal function [132,133]. The possible mechanism underlying the beneficial, neuroprotective effects of elevated bHB concentrations involve the improvement of mitochondrial function and energy balance (with bHB serving as a ‘superfuel’) in neurons. These effects may be especially important since glucose infusion has been reported to increase oxidative stress and exert adverse effects [134]. Recovery from TBI is slowed down by the metabolic dysfunction that occurs in the injured brain, such as the decrease in glucose metabolism seen following (head/brain) trauma [135,136,137]. Experiments on rats revealed that this condition lasts from several days up to 2 weeks [136,138]. Providing exogenous energy sources alternative to glucose (such as ketone bodies) during this period may improve mitochondrial function and therefore protect neurons from apoptosis after ischemia and TBI episodes [139]. In fact, ketone body uptake by the brain is facilitated by neuropathic conditions, such as TBI, ischemia or hemorrhagic shock, due to rapid changes in expression of monocarboxylate transporters [133].

### 6.4. Anti-Inflammatory Actions of PPARα and Ketone Bodies

Ketone bodies, particularly bHB, also possess anti-inflammatory activity, reduce inflammatory cytokine production and inhibit inflammasomes in immuno-competent cells [140,141,142], which may further limit neuronal cell loss and facilitate tissue regeneration following injury. The neuroprotective potential of bHB led to formulation of KTX 0101, a bHB sodium salt, which has been clinically tested and used as a neuroprotectant for patients undergoing major surgical procedures, such as cardiopulmonary bypass [143].

Taking into account these positive effects of ketone bodies in CNS pathology, one could anticipate the potential for neuroprotective effects of modulated by PPARα, considering that this nuclear receptor is the main transcription factor involved in ketogenesis. As expected, activation of PPARα with various synthetic and natural agonists has also been reported to have high efficacy in the treatment of a broad range of neurological disorders including epilepsy, traumatic brain injury, AD or PD [118]. This beneficial action is likely to be associated with the pro-ketogenic activities of PPARα, but also with its anti-inflammatory properties. PPARα is ubiquitously active in neurons and actrocytes, where its expression is the highest among all three PPAR isotypes (PPARα, PPARβ/δ, PPARγ) [144,145,146,147].

In general, the anti-inflammatory action of PPARα is driven by repression of pro-inflammatory genes, including cyclooxygenase-2 (COX-2), a variety of cytokines (IL-6, IL-12, IL-23, IL-27), and the inhibition of nitric oxide production in various cells [148,149,150,151,152,153]. The underlying mechanism involves inhibition of NFκB- and AP-1-mediated transactivation [154,155,156,157]. Arachidonic acid derived inflammatory mediators, such as leukotrienes LTB4 and LTA4 are natural PPARα agonists [158]. Activated PPARα induces leukotriene degradation through β- and ω-oxidation, as well as microsomal hydroxylation, which was proposed as a mechanism of inflammation resolution in experimental models and in patients with infectious diseases [158,159,160,161]. Pharmacological ligands of PPARα, fenofibrate and gemfibrozil, reduce the clinical manifestation of experimental autoimmune encephalomyelitis in an animal model of multiple sclerosis, by suppressing pro-inflammatory responses in astrocytes [149,150]. PPARα activation by natural agonists, such as acylethanolamides, reduces β amyloid-induced inflammation in astrocytes, exerting neuroprotection in AD models [162,163]. In addition, fenofibrate treatment demonstrated excellent neuroprotective effects against cerebral ischemia-reperfusion injury through its anti-oxidant and anti-inflammatory activities [164], and also reduced memory and learning deficits in rats subjected to global cerebral ischemia [165]. A separate set of anti-inflammatory actions of PPARα might be associated with catabolism and elimination of fatty-acyl derivatives and neuro-inflammatory lipids in astrocytes, in addition to the production and release of neuroprotective ketone bodies by these cells [107].

## 7. Ketogenesis and Ketolysis in Cancer Cells

Normal neurons and glial cells efficiently metabolize ketone bodies and produce energy through ketolysis [83,166]. Extensive research performed on various tumor cell lines, including gliomas, suggests that they do not utilize ketone bodies as energy substrates, but rather as precursors for lipid synthesis, and further, that some lack the enzymatic machinery for ketone body metabolism [166,167,168,169,170,171,172]. Other reports demonstrate that cancer cells do express at least some ketolytic enzymes and retain the ability to metabolize ketone bodies [173,174,175,176]. In breast and prostate carcinomas, expression of ketolytic enzymes is even regarded as a prognostic marker that is associated with aggressive phenotypes [177,178]. However, some studies indicate that despite the presence of ketolytic enzymes, the ability of ketone body utilization is lost during progression of malignant transformation [179]. Benign epithelial prostate cells are capable of fatty acid β-oxidation and can be rescued by bHB addition during glucose starvation, whereas the more aggressive cells lose metabolic plasticity and cannot switch to bHB metabolism [179]. A very special situation has been described in case of hepatocellular carcinoma cells, which reexpress the ketolytic enzyme SCOT during serum starvation [180]. In healthy hepatocytes, SCOT is not expressed in order to prevent ketone body synthesis and catabolism in the same location. Reexpression of SCOT in hepatocellular carcinoma removes this limitation and provides these cells with a growth advantage in the state of nutrient deprivation. Apparently, the serum starved hepatocellular carcinoma cells show simultaneous synthesis and utilization of ketone bodies. Although ketogenic diet did not promote tumor growth in nude mice bearing human HCC tumors, the retrospective analysis of clinical samples from liver cancer patients showed a significant correlation between low SCOT expression levels and longer survival [181].

It is important to note that the expression of ketolytic enzymes does not directly correlate with real ketolytic capacity. Murad and colleagues demonstrated that SCOT, the key ketolytic enzyme, undergoes nitration on tyrosine residues during various inflammatory conditions, and that this modification significantly decreases its catalytic activity [182,183]. Therefore, chronic inflammation, which frequently takes place in the tumor microenvironment, may lead to protein nitration and limit utilization of ketone bodies by tumor cells.

Breast cancer cells retain the capacity to perform oxidative metabolism at a high rate and take advantage of ketolysis to support their growth and progression [184]. Still, induction of ketosis by caloric restriction can slow down disease progression even in breast, prostate and gastric carcinomas [185,186,187,188,189].

The ketogenic diet and its calorie restricted variant that augments fasting responses, have been proposed to support anti-glioma therapy [190,191]. Indeed, bHB and acetoacetate exerted cytotoxic and growth inhibitory action on various cancer cells including lymphoma, melanoma, neuroblastoma, kidney and thyroid cancer cells [192,193]. Relatively mild adverse effects associated with ketogenic diet make it an ideal option for an adjunct anti-cancer regimen or even sometimes the main line of treatment. This approach turned out to be successful in treating malignant gliomas in experimental animals [194,195,196,197,198], and more importantly, for brain tumor patients [199,200,201]. Some other studies, however, did not confirm this promising outcome [173], which could indicate that numerous factors control the ability of cells to utilize ketone bodies in vivo and that sensitivity to their actions could depend on a particular cancer type, genetic mutation, or microenvironment conditions.

Under physiological conditions, the expression of ketogenic enzymes seems to be restricted to specialized cells of mature tissues. Recently, the expression of ketogenic enzymes has been also demonstrated in cancer cells of neuroectodermal origin, namely melanoma and glioblastoma [202,203]. The presence of HMGCL was detected in human melanoma and leukemia cells harboring the BRAF V600E mutation. In these cases, this enzyme induced intracellular accumulation of acetoacetate. Acetoacetate contributed to enhanced oncogenic activity of mutated BRAF and promoted MEK-Erk signaling and growth potential [202]. Conversely, stimulation of HMGCS2 expression and bHB production in murine melanoma B16 F10 by a PPARα ligand fenofibrate, was accompanied by cell growth arrest, energy stress and downregulation of enzymes involved in pentose phosphate pathway and lipid synthesis ([203] and unpublished data). Surprisingly, these effects of fenofibrate were PPARα independent.

Although the capability of performing ketolysis is a sign of metabolic flexibility of cancer cells and gives them a straightforward growth advantage during fasting, questions remain regarding the meaning and significance of ketogenesis in the transformed cells. Ketone body synthesis and secretion by tumor cells would provide additional positive nutritional stimuli for the surrounding normal cells and could reduce inflammation in the tumor microenvironment. If pharmaceuticals, such as fenofibrate, are able to reprogram cancer metabolism towards ketogenesis while simultaneously blocking proliferation, this might be beneficial from a therapeutic point of view, especially in conditions where inflammation and associated edema are particularly dangerous, as seen in gliomas and other brain tumors.

## 8. Concluding Remarks

Ketogenesis is an important evolutionary achievement of mammals, especially primates. PPARα receptors are an indispensable element of the cellular nutrient-sensing system and the chief regulators of the ketogenic gene expression program. Utilization of ketone bodies as a source of energy is a sign of metabolic flexibility, which is necessary for cell survival during fasting; however, ketone bodies also confer cytoprotection independent from their role in modulating energy potential. The gradual accumulation of experimental data will allow us to gain a better understanding of the role of ketone bodies in both physiological and pathological circumstances, and may open up new opportunities for their therapeutic application against metabolic and inflammatory diseases, as well as cancer.

## Abbreviations

ACAT1	Acetoacetyl-Coa Thiolase 1
ACC	Acetyl-CoA carboxylase
AD	Alzheimer’s disease
AMPK	AMP-activated kinase
BCAA	Branched chain amino acids
BCKD	Branched keto acid dehydrogenase
BDH	β-Hydroxybutyrate dehydrogenase
bHB	β-Hydroxy butyrate
CNS	Central nervous system
COUP-TF	Chicken ovoalbumin-upstream promoter transcription factor
COX-2	Cyclooxygenase 2
CPT1	Carnitine palmitoyltransferase 1
CREB	cAMP responsive element binding protein
FGF21	Fibroblast growth factor 21
GABA	γ amino butyric acid
HMGCL	3-Hydroxy-3-methylglutaryl-CoA lyase
HMGCR	3-Hydroxy-3-methylglutaryl-CoA reductase
HMGCS2	3-Hydroxy-3-methylglutaryl-CoA synthetase
HNF4	Hepatocyte nuclear factor 4
LCAD	Long chain acyl-CoA dehydrogenase
MCAD	Medium chain acyl-CoA dehydrogenase
MCT1	Monocarboxylate transporter 1
mTOR	Mammalian/mechanistic target of rapamycin
mTORC1	mTOR complex 1
NCoR	Nuclear coactivator repressor
NEFA	Nonesterified fatty acids
PD	Parkinson’s disease
PGC-1α	PPARγ coactivator 1α
PPAR	Peroxisome proliferator activated receptor
PPRE	Peroxisome proliferator response element
PUFA	Polyunsaturated fatty acid
ROR	Retinoid orphan receptor
SCOT	Succinyl-CoA:3-ketoacid-CoA transferase
TBI	Traumatic brain injury
TCA	Tricarboxylic acid cycle
TSC2	Tuberous sclerosis complex 2
